# Inflammatory Markers in Middle-Aged Obese Subjects: Does Obstructive Sleep Apnea Syndrome Play a Role?

**DOI:** 10.1155/2010/675320

**Published:** 2010-06-08

**Authors:** Paschalis Steiropoulos, Nikolaos Papanas, Evangelia Nena, Maria Antoniadou, Evangelia Serasli, Sophia Papoti, Olga Hatzizisi, Georgios Kyriazis, Argyris Tzouvelekis, Efstratios Maltezos, Venetia Tsara, Demosthenes Bouros

**Affiliations:** ^1^Department of Pneumonology, Medical School, Democritus University of Thrace, 68100 Alexandroupolis, Greece; ^2^Sleep Unit, Second Chest Department, General Hospital “George Papanikolaou”, 57010 Thessaloniki, Greece; ^3^Outpatient Clinic of Obesity, Diabetes and Metabolism, Second Department of Internal Medicine, Democritus University of Thrace, 68100 Alexandroupolis, Greece; ^4^Immunology Laboratory, General Hospital “George Papanikolaou”, 57010 Thessaloniki, Greece

## Abstract

*Background*. Obstructive Sleep Apnea Syndrome (OSAS) is associated with inflammation, but obesity may be a confounding factor. Thus, the aim of this study was to explore differences in serum levels of inflammation markers between obese individuals with or without OSAS. *Methods*. Healthy individuals (*n* = 61) from an outpatient obesity clinic were examined by polysomnography and blood analysis, for measurement of TNF-*α*, IL-6, CRP, and fibrinogen levels. According to Apnea-Hypopnea Index (AHI), participants were divided into two BMI-matched groups: controls (AHI < 15/h, *n* = 23) and OSAS patients (AHI ≥ 15/h, *n* = 38). *Results*. OSAS patients had significantly higher TNF-*α* levels (*P* < .001) while no other difference in the examined inflammation markers was recorded between groups. Overall, TNF-*α* levels were correlated with neck circumference (*P* < .001), AHI (*P* = .002), and Oxygen Desaturation Index (*P* = .002). *Conclusions*. Obese OSAS patients have elevated TNF-*α* levels compared to BMI-matched controls, suggesting a role of OSAS in promoting inflammation, possibly mediated by TNF-a.

## 1. Introduction

Obstructive Sleep Apnea Syndrome (OSAS) is a common disorder, known to affect about 4% of middle-aged men and 2% of middle-aged women [1]. Patients exhibit repetitive episodes of partial or complete obstruction of the upper airway during sleep, ultimately leading to increased respiratory effort, oxyhemoglobin desaturation, sleep fragmentation, and excessive daytime sleepiness. Increasing evidence suggests that OSAS is associated with hypertension and other cardiovascular diseases, metabolic derangement, and impaired glucose tolerance [2].

Obesity is not only a well-established risk factor for OSAS [1, 3–6] but also a proinflammatory state [7]. In contrast to earlier theories which considered the adipose tissue as a sole energy depot, current data demonstrate that it is an active endocrine organ, releasing a number of bioactive mediators (adipokines) that modulate blood pressure, lipid- and glucose-metabolism, atherosclerosis, and inflammation [7–9]. Indeed, macrophages of the adipose tissue secrete proinflammatory cytokines such as Tumor Necrosis Factor-*α* (TNF-*α*) and Interleukin-6 (IL-6) [7, 10].

Similarly, inflammation is one of the postulated links between OSAS and increased cardiovascular morbidity [2]. Indeed, the proinflammatory transcription factor NF-*κ*B is upregulated in OSAS. This is mediated by the alterations between hypoxia and reoxygenation, along with sleep deprivation. NF-*κ*B plays a key role in inflammatory responses, regulating the expression of inflammatory genes [11]. So far, previous studies, as reviewed by Arnardottir et al. [12], have recruited subjects from sleep laboratories, attempting to establish a link between OSAS and inflammation by focusing on the markers TNF-*α*, IL-6, and C-reactive protein (CRP). However, the contribution of obesity per se in this inflammatory activity has not been adequately determined [12]. 

Given that systemic inflammation has been demonstrated in both obesity and OSAS, there may be a possible interaction between them, rendering extremely difficult the distinction between inflammatory processes attributed to either of these two conditions. Therefore, the present study aimed to contribute to the clarification of such issues. Specifically, it attempted to explore the potential differences in four well-established serum inflammation markers (TNF-*α*, IL-6, CRP, and fibrinogen) between otherwise healthy, obese subjects with OSAS and their nonapneic obese counterparts.

## 2. Methods

### 2.1. Subjects

The present study included sixty-one (50 males and 11 females) subjects. These were consecutively recruited from the Outpatient Clinic of Obesity, Diabetes and Metabolism and had consented to be referred for sleep evaluation. None of them had been previously examined or had received treatment for obstructive sleep apnea. Body Mass Index (BMI) was calculated according to the formula BMI = Weight (in kilograms)/[Height (in meters)]^2^. All participants had a BMI exceeding 30 kg/m^2^.

Exclusion criteria were as follows: known inflammatory or other chronic disease, diabetes mellitus, cardiovascular or cerebrovascular, liver, or endocrine disease, hypertension, chronic use of medication, and smoking. Infection occurring at the time of the examination was an additional exclusion criterion. The study was approved by the institutional ethics committee and all participants had given their informed consent.

### 2.2. Study Design

#### 2.2.1. Initial Assessment

Medical history was recorded, and physical examination was performed. Anthropometrical data [age, sex, BMI, neck, waist and hip circumference, and waist-to-hip ratio (WHR)] along with daytime habits were recorded. Neck circumference was measured at the cricothyreoid level, waist circumference in the middle between the 12th rib and the iliac crest, and hip circumference at the level of great trochander by a measure tape. Blood pressure was recorded as the average of three repeated measurements in a seated position by an electronic sphygmomanometer adapted to arm circumference. Sleepiness was evaluated by the Greek version of Epworth Sleepiness Scale (ESS) [13].

#### 2.2.2. Polysomnography (PSG)

All subjects underwent an attended overnight polysomnography (Somnologica 3.1; Flaga; Reykjavik, Iceland) using a standard montage of electroencephalogram (EEG), electrooculogram (EOG), electromyogram (EMG), and electrocardiogram (ECG) signals together with pulse oximetry and airflow, detected using combined oronasal thermistors. Thoracic cage and abdominal motion were recorded by inductive plethysmography. Polysomnography was conducted between 22.00 and 06.00 hours. Apneas, hypopneas, and EEG recordings were manually scored according to standard criteria [14]. Apnea Hypopnea Index (AHI) was calculated by dividing the total number of apneas and hypopneas to polysomnographically recorded sleep time, while Oxygen Desaturation Index (ODI) was calculated by dividing the total number of oxyhaemoglobin desaturations ≥3% to polysomnographically recorded sleep time.

Two groups were formed, based on AHI in the polysomnographic examination: OSAS patients (AHI ≥ 15/hour; *n* = 38) and controls (AHI < 15/hour; *n* = 23).

#### 2.2.3. Measurement of Cytokines and Biochemical Analysis

Blood samples were collected between 8 and 9 AM following the polysomnographic examination, while participants were in a fasting state. After blood collection, serum was frozen in aliquots at −80°C immediately after centrifugation (4°C, 1600 g for 15 minutes). TNF-*α* and IL-6 levels were detected with quantitative sandwich enzyme immunoassay technique (R&D Systems, Minneapolis, USA). Minimum detectable doses of TNF-*α* and IL-6 were 1.6 pg/mL and 0.7 pg/mL, respectively. High-sensitivity CRP (CRP) was measured by nephelometric method in an image analyzer (Beckmann Coulter; California, USA). Fibrinogen levels were measured by clotting method using a Thrombolyzer B.E. (Behnk Elektronik GmbH) analyzer.

#### 2.2.4. Statistical Analysis

All continuous variables were checked for normality (Kolmogorov-Smirnov test). Descriptive results for continuous variables are expressed as mean ± SD. Differences between individuals with and without OSAS were examined with independent samples *t*-test or Mann-Whitney test, and correlations were explored with Pearson's or Spearman correlation, respectively, depending on the normality of data distribution. The reported *P*-values are two tailed. Significance was defined at the 5% level (*P* < .05). Analysis was performed using SPSS v.15.0 (SPSS Inc. Chicago, IL).

## 3. Results

Anthropometric and sleep characteristics of all subjects, as well as the comparison between the two groups (OSAS patients and controls), are presented in Table 1. The two groups were matched in terms of BMI, waist circumference and WHR; however, in OSAS patients a significantly greater mean neck circumference was observed. Indices of lung function, that is, spirometry and arterial blood gases' analysis, were within the normal range in all participants, and blood pressure measurements were below 140/90 mm Hg. As expected, characteristics of respiratory function during sleep in the OSAS group were obviously worse in comparison to the control group. 

**Figure 1:**
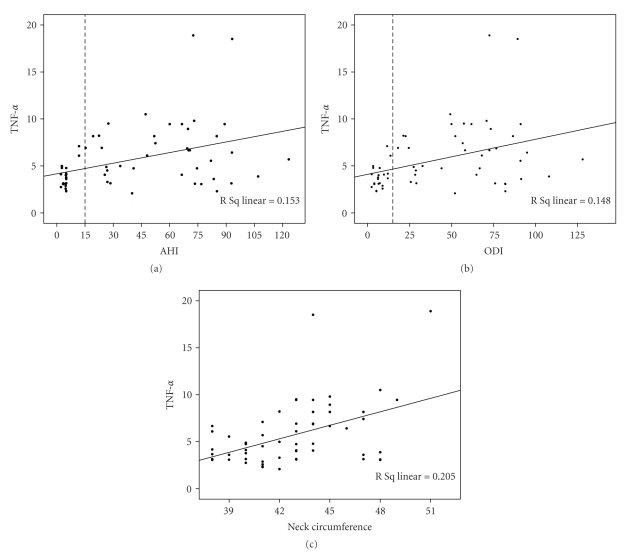
Association between TNF-*α* levels and AHI (a), ODI (b), and neck circumference (c) in the study population.

OSAS patients had significantly higher levels of TNF-*α* while no difference was detected between the two groups in levels of CRP, IL-6 and fibrinogen (Table 2). Overall, levels of TNF-*α* were significantly positively correlated with neck circumference (*r* = 0.452, *P* < .001), AHI (*r* = 0.391, *P* = .002), and ODI (*r* = 0.384, *P* = .002) (Figure 1). Interestingly, there was also a small but statistically significant negative correlation of CRP levels with average SpO_2_ (*r* = −0.252, *P* = .050) and minimum SpO_2_ (*r* = −0.256, *P* = .047) during sleep. No other correlation between the levels of the examined inflammation markers and anthropometric or sleep characteristics of the recruited subjects was observed.

## 4. Discussion

This study compared obese OSAS subjects with their non-apneic obese counterparts in terms of four established serum inflammation markers. In comparison to controls matched for BMI, WHR, and waist circumference, higher TNF-*α* levels were revealed in OSAS patients. TNF-*α* is an inflammatory cytokine that has been found elevated in patients with sleep apnea [15–17]. It is involved in sleep regulation [18, 19] and has been positively correlated with excessive daytime sleepiness, nocturnal sleep disturbance, and hypoxia [20]. Similar to our findings, Ciftci et al. [15] have reported increased TNF-*α* levels in the presence of OSAS, and this increase was independent of BMI. However, they studied only males, whom they recruited from a sleep disorders center, instead of an obesity clinic [15]. Elevation of TNF-*α* has also been observed by Minoguchi et al. [16], but, again, the comparison was between OSAS and obese subjects. Ryan et al. [17] have demonstrated higher TNF-*α* levels in subjects with than in those without OSAS, but they studied exclusively men and did not examine the impact of obesity on TNF-*α* elevation. Our study differs from the previous works in two ways. First, we enrolled subjects from an outpatient clinic of obesity, diabetes, and metabolism. Secondly, all our subjects were obese. This enabled us to address (for the first time, to the best of our knowledge) the interplay between obesity and OSAS in the obesity clinic, by comparing OSAS subjects with their non-apneic counterparts. Hence, our new message is that TNF-*α* is increased in the presence of OSAS among obese subjects. 

By contrast, there was no difference between our two groups in the other three inflammatory markers, that is, CRP, IL-6, and fibrinogen. Prior research has failed to reach a definitive conclusion regarding levels of CRP or IL-6 in OSAS patients. Some authors have reported elevated serum CRP levels [21–24] while others have come to the opposite conclusion [25–28]. Similarly, levels of IL-6, the cytokine inducing the production of CRP, were found elevated in OSAS patients in numerous studies [15, 20, 24], but most of them were criticized for the small number of patient series, the lack of proper match between patients and controls in BMI, and the inclusion of patients with cardiovascular or metabolic disorders [29]. On the contrary, other works did not manage to establish a correlation between OSAS and IL-6 [17, 30]. Interestingly, continuous positive airway pressure therapy (CPAP) has been shown to reduce CRP in patients with adequate compliance to treatment [31]. It appears, therefore, that the role of CRP and IL-6 in OSAS is far from settled and that additional research is required to shed more light on this issue. In this endeavor for further elucidation of the role of CRP and IL-6 in OSAS, our report adds that these inflammatory markers are not increased in obese OSAS subjects as compared to their non-apneic peers. 

Fibrinogen levels are increased in OSAS patients, according to previous workers [32–34], indicating a predisposition for coagulation and atherosclerosis. Conversely, the present study failed to demonstrate a difference in fibrinogen levels between our two groups of obese individuals, suggesting that the presence of OSAS probably does not play an important role in the upregulation of fibrinogen levels. Again, the discrepancy between our results and those of previous research may be, partly at least, explained by the different setting (patients from obesity clinic versus those from a sleep unit). Nonetheless, further large-scale studies to re-examine fibrinogen levels in OSAS would be appreciated. 

TNF-*α* levels showed a significant positive correlation with AHI and ODI. The latter corroborates the finding by Ryan et al. [17] where ODI was the strongest predictor of TNF-*α* levels, indicating the role of intermittent hypoxia and re-oxygenation in the pathogenesis of inflammation through the activation of NF-*κ*B, a transcription factor that has an important role in inflammatory responses [35]. An additional finding was the correlation between CRP levels and indices of nocturnal hypoxia, like average or minimum SpO_2_. Taken together, these results suggest that hypoxia, manifested as repetition of desaturations or as lower levels of average or minimum SpO_2_, is probably the major contributor in the activation of inflammation in OSAS.

Moreover, a significantly greater neck circumference was noted in OSAS patients, despite the lack of difference in other characteristics of obesity, like BMI or WHR. This observation is common in OSAS patients and many authors have emphasized the association between neck circumference and apneas [36]. Additionally, a positive association between TNF-*α* levels and neck circumference was observed in our study population while we did not manage to establish a correlation between this marker and other indices of obesity.

The limitations of this study may be outlined as follows. First, the majority of recruited subjects were male. Therefore, some reservation is needed in applying our findings to females. Additionally, although waist circumference is increasingly recognized to be the best indicator for the degree of visceral adiposity, we did not employ MRI to measure directly fat amount and fat distribution, as this would substantially increase the cost of the study [37, 38]. 

The present work may have the following practical implications. Among obese subjects, those with new diagnosis of OSAS exhibit more pronounced inflammation, as evidenced by the increased levels of TNF-*α*. Moreover, it is worth noting that application of CPAP has already been shown to reduce levels of this cytokine in overt OSAS [39]. Hence, it is conceivable that inflammation, in general, and TNF-*α*, in particular, may prove a useful target for therapeutic intervention in obese patients. Vice versa, given that repeated episodes of hypoxia were identified as a major contributor in the activation of inflammation among newly diagnosed OSAS patients in this study, one could also argue that early evaluation of the obese patient for the presence of OSAS might be anticipated to help towards mitigation of chronic inflammation. Certainly, the interaction between inflammation and OSAS in obese patients is complicated and merits further study.

## 5. Conclusions

The present study shows that, among obese subjects, TNF-*α* levels are increased in the presence of OSAS, as compared to non-apneic BMI-matched controls. Additionally, TNF-*α* levels are associated with respiratory disturbance during sleep, as depicted by AHI and ODI as well as larger neck circumference, a common feature of OSAS patients. Our results suggest a role for OSAS in promoting inflammation among obese subjects, possibly mediated by TNF-*α*. Further work is now awaited to confirm these findings in larger series, as well as to investigate the potential role of early therapeutic intervention in the obesity clinic to alleviate chronic inflammation.

## Figures and Tables

**Table 1:** Comparison of anthropometric and sleep characteristics between the two groups.

	Total	Controls	OSAS	*P*-value
	(*n* = 61)	(*n* = 23)	(*n* = 38)	
Gender (M/F)	50/11	17/6	33/5	.353
Age (years)	44.9 ± 9.2	43.7 ± 6.7	45.5 ± 10.5	.420
BMI (kg/m^2^)	35.7 ± 6.3	34.5 ± 3.7	36.4 ± 7.4	.577
Neck circumference (cm)	42.8 ± 3.1	40.5 ± 2	44.1 ± 3	<**.001**
Waist circumference (cm)	117 ± 9.7	114.7 ± 7.9	118.4 ± 10.5	.147
Hip circumference (cm)	118.7 ± 13.7	114.7 ± 8.2	121.2 ± 15.7	.140
WHR	0.99 ± 0.05	1 ± 0.03	0.98 ± 0.06	.169
Systolic BP (mmHg)	131.1 ± 7	131.2 ± 6.4	131.1 ± 7.4	.864
Diastolic BP (mmHg)	80.4 ± 7.9	80.2 ± 7.9	80.6 ± 7.9	.873
FEV_1 _(% pred)	91.1 ± 9.7	91.2 ± 8.9	91 ± 10.3	.939
FVC (% pred)	89.6 ± 10.2	89 ± 8.8	90 ± 11	.714
PaO_2_ (mmHg)	82.4 ± 6.8	82.7 ± 5.8	82.2 ± 7.5	.786
PaCO_2_ (mmHg)	38.4 ± 2.5	38.6 ± 1.3	38.2 ± 3	.428
pH	7.40 ± 0.02	7.40 ± 0.01	7.41 ± 0.02	.943
ESS	9.9 ± 6.2	6.6 ± 4.6	11.9 ± 6.2	<**.001**
AHI (/hour)	40 ± 34.5	5.3 ± 3.2	61 ± 27	<**.001**
ODI (/hour)	41.9 ± 34.2	7.3 ± 4	62.9 ± 26.4	<**.001**
avSpO_2_ (%)	90.8 ± 4.6	93.4 ± 1.4	89.3 ± 5.2	<**.001**
minSpO_2_ (%)	75.7 ± 11.1	84.4 ± 3.1	70.4 ± 10.8	<**.001**
*t* < 90 (% TST)	23.6 ± 27.8	3.3 ± 5	36.3 ± 28.7	<**.001**

**Table 2:** Comparison of levels of the examined inflammatory markers in the two groups.

	Total	Controls	OSAS	*P*-value
	(*n* = 61)	(*n* = 23)	(*n* = 38)	
TNF-*α* (pg/mL)	5.67 ± 3.32	3.94 ± 1.34	6.72 ± 3.72	<**.001**
CRP (mg/dL)	0.52 ± 0.48	0.47 ± 0.5	0.55 ± 0.47	.125
IL-6 (pg/mL)	2.66 ± 1.19	2.36 ± 1.41	2.73 ± 1.14	.465
Fibrinogen (g/L)	2.63 ± 0.75	2.7 ± 0.65	2.59 ± 0.81	.616

## Abbreviations

BMI: Body Mass Index
WHR: Waist to Hip ratio
BP: Blood pressure
FEV_1_: Forced Expiratory Volume in 1st second
FVC: Forced Vital Capacity
ESS: Epworth Sleepiness Scale
AHI: Apnea Hypopnea Index
ODI: Oxygen Desaturation Index
avSpO_2_: Average saturation during sleep (in pulse oxymetry)
minSpO_2_: Minimum saturation during sleep (in pulse oxymetry)
*t* < 90: Sleep time with SpO_2 _<90%
TST: Total sleep time.
